# Supplementary material for: Interactions among Escovopsis, Antagonistic Microfungi Associated with the Fungus-Growing Ant Symbiosis
Source: J Fungi (Basel). 2021 Nov 25;7(12):1007. doi: 10.3390/jof7121007 (PMC8703566; doi:10.3390/jof7121007)
Supplement: Supplementary file 1 [file jof-07-01007-s001.zip › jof-1388340-supplementary.pdf]

Table S1. Metadata for strains included in phylogenetic analyses and interactions experiments. Colony and sequence information used in this study

No.	Ant Host	Colony Code	Location (Province)	Fungal Strain ID	LSU GenBank accession number
1	*Mycetomoellerius zeteki* 178 COL	20141101-07 YG	Gamboa (Colon)	M27	MZ959192
2	*Mycetomoellerius zeteki* 175 COL	20150527-15 CMV	Gamboa (Colon)	M5	MZ959193
3	*Mycetomoellerius zeteki* 174 COL	20150610-17 CMY	Gamboa (Colon)	M17	MZ959194
4	*Mycetomoellerius zeteki* 173 COL	20141128-11 CGV	Gamboa (Colon)	M11	MZ959195
5	*Mycetomoellerius zeteki* 172 COL	20150612-24 CMY	Gamboa (Colon)	M2	MZ959196
6	*Mycetomoellerius zeteki* 168 COL	20141101-08 VYCG	Gamboa (Colon)	M26	MZ959197
7	*Mycetomoellerius zeteki* 167 COL	BP20130627-TB	Gamboa (Colon)	M25	MZ959198
8	*Mycetomoellerius zeteki* 166 COL	20150610-16 CMY	Gamboa (Colon)	M24	MZ959199
9	*Mycetomoellerius zeteki* 164 COL	20150210-14 YCH	Gamboa (Colon)	M20	MZ959200
10	*Mycetomoellerius zeteki* 163 COL	20150204-13 CGY	Gamboa (Colon)	M19	MZ959201
11	*Mycetomoellerius zeteki* 162 COL	20150612-22 CMY	Gamboa (Colon)	M18	MZ959202
12	*Mycetomoellerius zeteki* 161 COL	20150612-21 CMY	Gamboa (Colon)	M16	MZ959203
13	*Mycetomoellerius zeteki* 160 COL	20141107-10 YG	Gamboa (Colon)	M15	MZ959204
14	*Mycetomoellerius zeteki* 159 COL	20150612-19 CMY	Gamboa (Colon)	M14	MZ959205
15	*Mycetomoellerius zeteki* 158 COL	20150610-18 CMY	Gamboa (Colon)	M13	MZ959206
16	*Mycetomoellerius zeteki 157 COL*	20150123-12 VB	Gamboa (Colon)	M12	MZ959207
17	*Mycetomoellerius zeteki* 155 COL	20150612-20 CMY	Gamboa (Colon)	M21	MZ959208
18	*Mycetomoellerius zeteki* 153 COL	20150612-23 CMY	Gamboa (Colon)	M3	MZ959209
19	*Mycetomoellerius zeteki* 152 COL	20150625-25 MV	Gamboa (Colon)	M8B	MZ959210
20	*Paratrachymyrmex cornetzi* 156 COL	20160626-03 CS	Gamboa (Colon)	P10	MZ959211
21	*Paratrachymyrmex cornetzi* 151 COL	20160626-07 CS	Gamboa (Colon)	P6	MZ959212
22	*Paratrachymyrmex cornetzi* 148 COL	20141101-09 VYCG	Gamboa (Colon)	P7	MZ959213
23	*Paratrachymyrmex cornetzi* 146 COL	20141101-06 VYCG	Gamboa (Colon)	P4	MZ959214
24	*Cyphomyrmex muelleri* 225 COL	20140902-14 HFM	Gamboa (Colon)	C14	MZ959215
25	*Cyphomyrmex* sp. 219 COC	20160329-09 YM	El Cope (Cocle)	CN6	MZ959216
26	*Cyphomyrmex* sp.217 COC	20160329-07 GMY	El Cope (Cocle)	CN4	MZ959217
27	*Myrmicocrypta ednaella* 234 COL	20141113-06 HFM	Gamboa (Colon)	M5	MZ959218
28	*Myrmicocrypta ednaella* 232 COL	201305-04MYR EG	Gamboa (Colon)	M4a	MZ959219
29	*Myrmicocrypta ednaella* 228 COL	20161130-02 GB	Gamboa (Colon)	M6b	MZ959220
30	*Myrmicocrypta ednaella* 230 COL	201305-03MYR EG	Gamboa (Colon)	M3a	MZ959221
31	*Myrmicocrypta ednaella* 229 COL	201305-02MYR EG	Gamboa (Colon)	M2	MZ959222
32	*Myrmicocrypta ednaella* 231 COL	201305-01MYR EG	Gamboa (Colon)	M1	MZ959223
33	*Mycocepurus smithii* 238 COL	2013-17732-EG	Gamboa (Colon)	238	MZ959224
34	*Mycocepurus smithii* 237 COL	2013-17731-EG	Gamboa (Colon)	237	MZ959225
35	*Cyphomyrmex longiscapus* 206 COL	BP20130712-47 PLR	Gamboa (Colon)	C18	MZ959226
36	*Cyphomyrmex muelleri* 205 COL	20140922-35 VB	Gamboa (Colon)	C35	MZ959227
37	*Apterostigma pilosum* 01 COL	LA20130722-71 Q	Gamboa (Colon)	AY3	MZ959228
38	*Apterostigma pilosum* 04 COL	LA20130617-Y	Gamboa (Colon)	AY4	MZ959229
39	*Apterostigma pilosum* 02 COL	LA20130722-B2	Gamboa (Colon)	AB2.2	MZ959230
40	*Apterostigma pilosum* 05 COL	LA20130723-69 Q	Gamboa (Colon)	AY2	MZ959231
41	*Apterostigma pilosum* 03 COL	LA20130723-B1	Gamboa (Colon)	AB1.3	MZ959232
42	*Apterostigma pilosum* 02 COL	LA20130722-B2	Gamboa (Colon)	AY	MZ959233
43	*Apterostigma pilosum* 25 COL	LA20130722-55 Q	Gamboa (Colon)	AB3.1	MZ959234
44	*Acromyrmex octospinosus* 134 COL	2014113-04 VB	Gamboa (Colon)	AC4	MZ959235
45	*Acromyrmex octospinosus* 135 COL	20141113-05 VB	Gamboa (Colon)	AC5	MZ959236
46	*Acromyrmex octospinosus* 127 COL	20150123-10 VB	Gamboa (Colon)	AC10	MZ959237
47	*Acromyrmex octospinosus* 115 COL	20150521-15 VB	Gamboa (Colon)	AC35	MZ959238
48	*Acromyrmex octospinosus* 113 COL	20150123-11 VB	Gamboa (Colon)	AC11	MZ959239
49	*Acromyrmex echinatior* 145 WP	20140930-03	Chame (West Panama)	AC3	MZ959240
50	*Apterostigma dentigerum* 06 COL	BP20130806-87	Gamboa (Colon)	AY7	MZ959241
51	*Apterostigma dentigerum* 09 COL	BP20130806-89	Gamboa (Colon)	AY9	MZ959242
52	*Apterostigma dentigerum* 002COL	BP20130705-39 CT	Gamboa (Colon)	Whitish	MZ959243

53	*Apterostigma dentigerum* 07COL	BP20130806-88	Gamboa (Colon)	AY8	MZ959244
54	*Apterostigma dentigerum* 01COL	T20130604-02 CT	Gamboa (Colon)	AB1.2	MZ959245
55	*Apterostigma dentigerum*	LA20130722-78	Gamboa (Colon)	AB2.7	MZ959246
56	*Apterostigma dentigerum* 01 COL	BP20130618-19	Gamboa (Colon)	AY6	MZ959247
57	*Apterostigma dentigerum* 05 COL	BP20130719-B3	Gamboa (Colon)	AB3.5	MZ959248
58	*Atta colombica* 60 COL	20150615-26 MV	Gamboa (Colon)	AT 30	MZ959249
59	*Atta colombica* 59 COL	20150615-25 MV	Gamboa (Colon)	AT49	MZ959250
60	*Atta colombica* 106 COC	YM140216-06	El Cope (Cocle)	ATCOL7	MZ959251
61	*Atta colombica* 105 COC	YM140216-05	El Cope (Cocle)	ATCOL5	MZ959252
62	*Atta colombica* 104 COC	YM140216-04	El Cope (Cocle)	ATCOL4	MZ959253
63	*Atta colombica* 103 COC	YM140216-03	El Cope (Cocle)	ATCOL3	MZ959254
64	*Atta colombica* 88 COL	20150613-19 MV	Gamboa (Colon)	AT44	MZ959255
65	*Atta colombica* 81 COL	20150527-21 CMV	Gamboa (Colon)	AT41	MZ959256
66	*Atta colombica* 79 COL	20141030-09 CHG	Gamboa (Colon)	AT19B	MZ959257
67	*Atta colombica* 77 COL	20150618-27 MV	Gamboa (Colon)	AT43	MZ959258
68	*Atta colombica* 76 COL	20141028-16 CHG	Gamboa (Colon)	AT6	MZ959259
69	*Atta colombica* 63 COL	20141030-03 CHG	Gamboa (Colon)	AT19	MZ959260
70	*Atta colombica* 62 COL	20141030-02 CHG	Gamboa (Colon)	AT16	MZ959261
71	*Atta colombica* 61 COL	20141028-11 CHG	Gamboa (Colon)	AT11	MZ959262
72	*Apterostigma collare* 43 BCT	20160624-13 YA	Changuinola (Bocas del Toro)	BC17	MZ959263
73	*Apterostigma collare* 41 BCT	20160624-14 YA	Changuinola (Bocas del Toro)	BC14	MZ959264
74	*Apterostigma collare* 40 BCT	20160624-04 YA	Changuinola (Bocas del Toro)	BC13	MZ959265
75	*Apterostigma collare* 39 BCT	20160624-12 YA	Changuinola (Bocas del Toro)	BC12	MZ959266
76	*Apterostigma collare* 37 BCT	20160624-17 YA	Changuinola (Bocas del Toro)	BC10	MZ959267
77	*Apterostigma collare* 34 BCT	20160624-08 YA	Changuinola (Bocas del Toro)	BC7	MZ959268
78	*Apterostigma collare* 33 BCT	20160624-06 YA	Changuinola (Bocas del Toro)	BC6	MZ959269
79	*Apterostigma collare*	20160624-16 YA	Changuinola (Bocas del Toro)	BC3	MZ959270
80	*Apterostigma collare* 28 BCT	20160624-10 YA	Changuinola (Bocas del Toro)	BC1	MZ959271
81	*Atta cephalotes* 99 COC	YM110216-09	El Cope (Cocle)	ATCEPH4	MZ959272
82	*Atta cephalotes* 93 COC	YM110216- 03	El Cope (Cocle)	ATCEPH5	MZ959273
83	*Atta cephalotes* 92 COC	YM110216-02	El Cope (Cocle)	ATCEPH7	MZ959274
84	*Apterostigma auriculatum* 47 DRN	20160528-07YC	Chucanti (Darien)	DRN4 A	MZ959275
85	*Apterostigma auriculatum* 56 DRN	20160528-09 YC	Chucanti (Darien)	DRN10	MZ959276
86	*Apterostigma auriculatum* 54 DRN	20160529-14 YC	Chucanti (Darien)	DRN8 B	MZ959277
87	*Apterostigma auriculatum* 51 DRN	20160529-11 YC	Chucanti (Darien)	DRN6	MZ959278
88	*Apterostigma auriculatum* 48 DRN	20160528-08YC	Chucanti (Darien)	DRN4 B	MZ959279
89	*Apterostigma auriculatum* 46 DRN	20160529-06 YC	Chucanti (Darien)	DRN3	MZ959280
90	*Apterostigma auriculatum* 45 DRN	20160527-05 YC	Chucanti (Darien)	DRN2	MZ959281
91	*Apterostigma auriculatum* 44 DRN	20160527-04 YC	Chucanti (Darien)	DRN1	MZ959282
92	*Escovopsis microspora*				KF293284
93	*Escovopsis weberi*				AY172606
94	*Escovopsis moelleri*				JQ855715
95	*Escovopsis aspergilloides*				KF293283
96	*Escovopsis lentecrescens*				JQ855717
97	*Escovopsis primorosea*				KU298290
98	*Escovopsis catenulata*				KU298285
99	*Escovopsis longivesica*				KU298296
100	*Escovopsis kreiselli*				KJ808765
101	*Escovopsis clavatus*				MH715110
102	*Escovopsis multiformis*				MH715105
103	*Escovopsis trichodermoides*				MH715102
104	*Escovopsioides nivea*				JQ855716
105	*Hypomyces asterophorum*				AJ583469
106	*Hypomyces protrusum*				FN859414
107	*Hypomyces samuelsii*				FN859451
108	*Hypomyces semicirculare*				FN859417
109	*Hypocrea lutea*				JN941458

No.	Ant Host	Colony Code	Location (Province)	Fungal Strain ID	ITS GenBank accession number
1	*Mycetomoellerius zeteki* 167 COL	BP20130627-TB	Gamboa (Colon)	M25	MZ964338
2	*Atta colombica* 12 COL	20141030-012 CYG I	Gamboa (Colon)	AT24	MZ964339
3	*Atta colombica* 13 COL	20141028-03 CHG	Gamboa (Colon)	AT3	MZ964340
4	*Atta colombica* 01 COL	20141028-01 CHG	Gamboa (Colon)	AT1	MZ964341
5	*Atta colombica* 19 COL	20141028-09 CHG	Gamboa (Colon)	AT9	MZ964342
6	*Atta colombica* 81 COL	20150527-21 CMV	Gamboa (Colon)	AT41	MZ964343
7	*Atta cephalotes* 93 COC	YM110216- 03	El Cope (Cocle)	ATCEPH5	MZ964344
8	*Atta colombica* 77COL	20150618-27 MV	Gamboa (Colon)	AT43	MZ964345
9	*Atta colombica* 61 COL	20141028-11 CHG	Gamboa (Colon)	AT11	MZ964346
10	*Atta colombica* 16 DRN	20150330-16 HFM	Llano Carti (Darien)	AT16	MZ964347
11	*Atta colombica* 20 COL	20150331-20 HFM	Gamboa (Colon)	AT47	MZ964348
12	*Atta colombica* 17 DRN	20150330-17 HFM	Llano Carti (Darien)		MZ964349
13	*Sericomyrmex amabilis* 02 COL	20141108-08 GY	Gamboa (Colon)	S2	MZ964350
14	*Atta cephalotes* 92 COC	YM110216-02	El Cope (Cocle)	ATCEPH7	MZ964351
15	*Atta colombica*	20141027-02 CHG	Gamboa (Colon)	N2	MZ964352
16	*Atta colombica* 15 DRN	20150330-15 HFM	Llano Carti (Darien)	AT15	MZ964353
17	*Acromyrmex echinatior* 118 WP	20160603-01 YG	Chame (West Panama)	AC38	MZ964354
18	*Atta colombica* 105 COC	YM140216-05	El Cope (Cocle)	ATCOL5	MZ964355
19	*Atta sexdens* 56 COL	20150331-18 HFM	Donoso (Colon)	AT36	MZ964356
20	*Atta colombica* 109 COL	20141031-09 CHG	Gamboa (Colon)	AT45	MZ964357
21	*Acromyrmex echinatior* 146 WP	20150603-16 HFM	Chame (West Panama)	AC16	MZ964358
22	*Acromyrmex echinatior* 147 WP	20150603-17 HFM	Chame (West Panama)	AC17	MZ964359
23	*Acromyrmex octospinosus* 136 COL	20141113-06 VB	Gamboa (Colon)	AC6	MZ964360
24	*Acromyrmex echinatior* 144 CWP	20140930-02	Chame (West Panama)	AC2	MZ964361
25	*Atta sexdens*	20150331-19 HFM	Donoso (Colon)	AT46	MZ964362
26	*Atta colombica* 73 COL	20141030-011 YG	Gamboa (Colon)	AT22	MZ964363
27	*Acromyrmex echinatior* 143 WP	20140930-01	Chame (West Panama)	AC1	MZ964364
28	*Atta colombica* 88 COL	20150613-19 MV	Gamboa (Colon)	AT44	MZ964365
29	*Acromyrmex echinatior* 145 WP	20140930-03	Chame (West Panama)	AC3	MZ964366
30	*Acromyrmex octospinosus* 127 COL	20150123-10 VB	Gamboa (Colon)	AC10	MZ964367
31	*Mycetomoellerius zeteki* 001 COL	20150625-27 MV	Gamboa (Colon)	M27	MZ964368
32	*Paratrachymyrmex cornetzi* 151 COL	20160626-07 CS	Gamboa (Colon)	P6	MZ964369
33	*Sericomyrmex amabilis* 01 COL	20141028-04	Gamboa (Colon)	S1	MZ964370
34	*Trachymyrmex* sp.10	20161130 GB	Gamboa (Colon)	Thz3	MZ964371
35	*Apterostigma dentigerum* 01COL	T20130604-02CT	Gamboa (Colon)	AB1.2	MZ964372
36	*Apterostigma pilosum* 03 COL	LA20130723-B1	Gamboa (Colon)	AB1.3	MZ964373
37	*Apterostigma pilosum* 02 COL	LA20130722-B2	Gamboa (Colon)	AB2.2	MZ964374
38	*Apterostigma dentigerum* 07 COL	LA20130722-47	Gamboa (Colon)	AB2.7	MZ964375
39	*Apterostigma dentigerum* 05 COL	BP20130719-B3	Gamboa (Colon)	AB3.5	MZ964376
40	*Apterostigma collare* 33 BCT	20160624-06 YA	Changuinola (Bocas del Toro)	BC6	MZ964377
41	*Cyphomyrmex* sp. 214 COC	20160216-04 YM	El Cope (Cocle)	CN1	MZ964378
42	*Cyphomyrmex* sp. 217 COC	20160329-07 GMY	El Cope (Cocle)	CN4	MZ964379
43	*Cyphomyrmex* sp. 219 COC	20160329-09 YM	El Cope (Cocle)	CN6	MZ964380
44	*Myrmicocrypta ednaella* 232 COL	201305-04MYR EG	Gamboa (Colon)	M4a	MZ964381
45	*Myrmicocrypta ednaella* 234 COL	20141113-06 HFM	Gamboa (Colon)	M5	MZ964382
46	*Cyphomyrmex muelleri* 225 COL	20140902-14 HFM	Gamboa (Colon)	C14	MZ964383
47	*Cyphomyrmex longiscapus* 226 COL	20150626- MV	Gamboa (Colon)	CY6	MZ964384
48	*Apterostigma collare* 42 BCT	20160624-11 YA	Changuinola (Bocas del Toro)	BC16	MZ964385
49	*Apterostigma auriculatum* 45 DRN	20160527-05 YC	Chucanti (Darien)	DRN2	MZ964386
50	*Apterostigma auriculatum* 48 DRN	20160528-08YC	Chucanti (Darien)	DRN4 B	MZ964387
51	*Apterostigma dentigerum* 001 COL	BP20130618-19	Gamboa (Colon)	AY6	MZ964388
52	*Apterostigma dentigerum* 02B COL	BP20130705-38CT	Gamboa (Colon)	AY8	MZ964389

53	*Apterostigma dentigerum* 002 COL	BP20130705-39CT	Gamboa (Colon)	Whitish	MZ964390
54	*Apterostigma auriculatum* 51 DRN	20160529-11 YC	Chucanti (Darien)	DRN6	MZ964391
55	*Apterostigma auriculatum* 54 DRN	20160529-14 YC	Chucanti (Darien)	DRN8 B	MZ964392
56	*Apterostigma pilosum* 004 COL	LA20130617-Y	Gamboa (Colon)	AY4	MZ964393
57	*Apterostigma auriculatum* 49 DRN	20160529-12 YC	Chucanti (Darien)	DRN5	MZ964394
58	*Apterostigma collare* 28 BCT	20160624-10 YA	Changuinola (Bocas del Toro)	BC1	MZ964395
59	*Apterostigma collare* 36 BCT	20160624-09 YA	Changuinola (Bocas del Toro)	BC9	MZ964396
60	*Apterostigma collare* 39 BCT	20160624-12 YA	Changuinola (Bocas del Toro)	BC12	MZ964397
61	*Apterostigma collare* 40 BCT	20160624-04 YA	Changuinola (Bocas del Toro)	BC13	MZ964398
62	*Mycocepurus smithii* 237 COL	2013-17731-EG	Gamboa (Colon)	237	MZ964399
63	*Mycocepurus smithii* 238 COL	2013-17732-EG	Gamboa (Colon)	238	MZ964400
64	*Apterostigma auriculatum* 46 DRN	20160529-06 YC	Chucanti (Darien)	DRN3	MZ964401
65	*Escovopsis weberi*				KF293286
66	*Escovopsis microspora*				JQ815076
67	*Escovopsis moelleri*				JQ815077
68	*Escovopsis aspergilloides*				KF293287
69	*Escovopsis clavatus*				MH715096
70	*Escovopsis multiformis*				MH715091
71	*Escovopsis lentecrescens*				JQ815079
72	*Escovopsis kreiselli*				NR155087
73	*Escovopsioides nivea*				JQ815078
74	*Escovopsis trichodermoides*				MH715088
75	*Hypomyces samuelsii*				FN859451
76	*Hypomyces protrusum*				FN859414
77	*Hypomyces asterophorum*				FN859395
78	*Hypomyces semicircularis*				NR121425

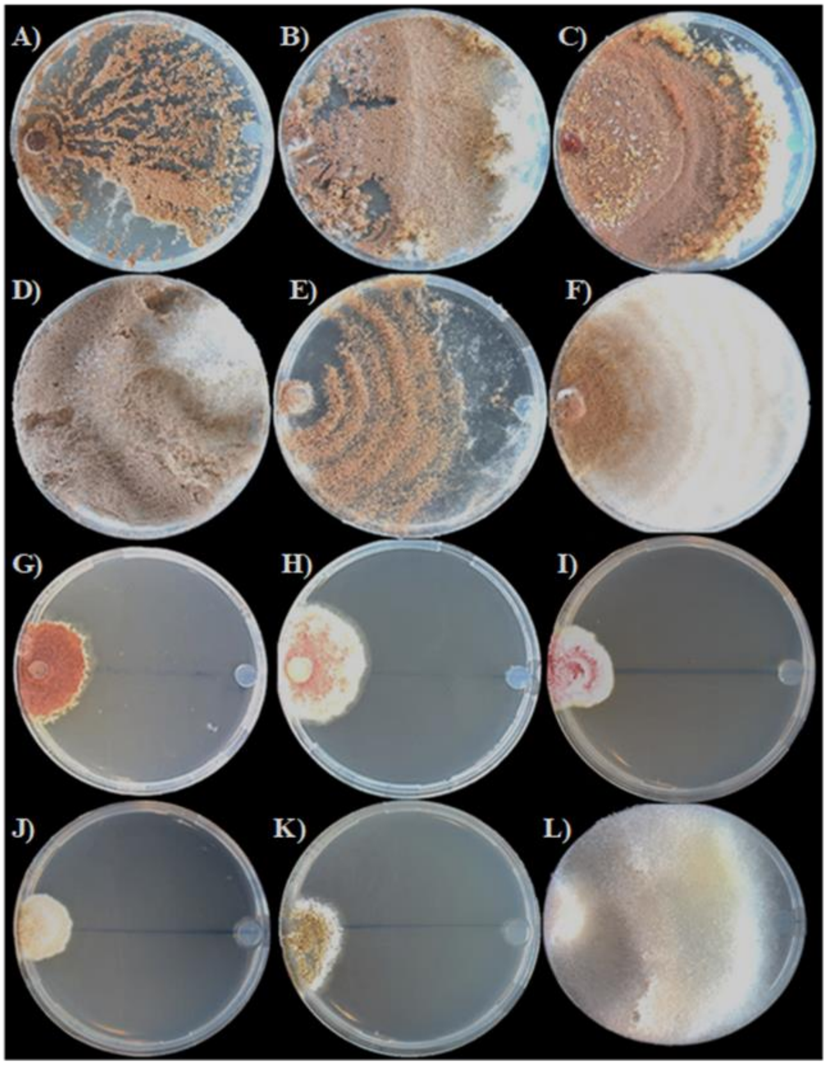

Figure S1. Intraclonal confrontation bioassays *Escovopsis* strains controls. Each of the 12 *Escovopsis* cultures was paired with a mycelium-free PDA agar block. (A) *Escovopsis* sp. from *At*. *colombica* colony; B) *Escovopsis* sp. from *At*. *cephalotes* colony; C) *Escovopsis* sp. from *At*. *sexdens* colony; D) *Escovopsis sp*. from *Par*. *cornetzi* colony; E) *Escovopsis* sp. from *Myc*. *zeteki* colony; F) *Escovopsis* sp. from *T*. *sp* 10 colony; G) *Escovopsis* sp. from *C*. *sp*. colony; H) *Escovopsis* sp. from C. *longiscapus* colony; I) *Escovopsis* sp. from *C*. *muelleri* colony; J) *Escovopsis* sp. from *Ap*. *auriculatum* colony; K) *Escovopsis* from *Ap*. *pilosum* colony, and L) *Escovopsis* sp. from *Ap*. *collare* colony.
